# Supplementary figures and images for: Atypical Relationships Between Neurofunctional Features of Print-Sound Integration and Reading Abilities in Chinese Children With Dyslexia
Source: Front Psychol. 2022 Jan 25;12:748644. doi: 10.3389/fpsyg.2021.748644 (PMC8822058; doi:10.3389/fpsyg.2021.748644)

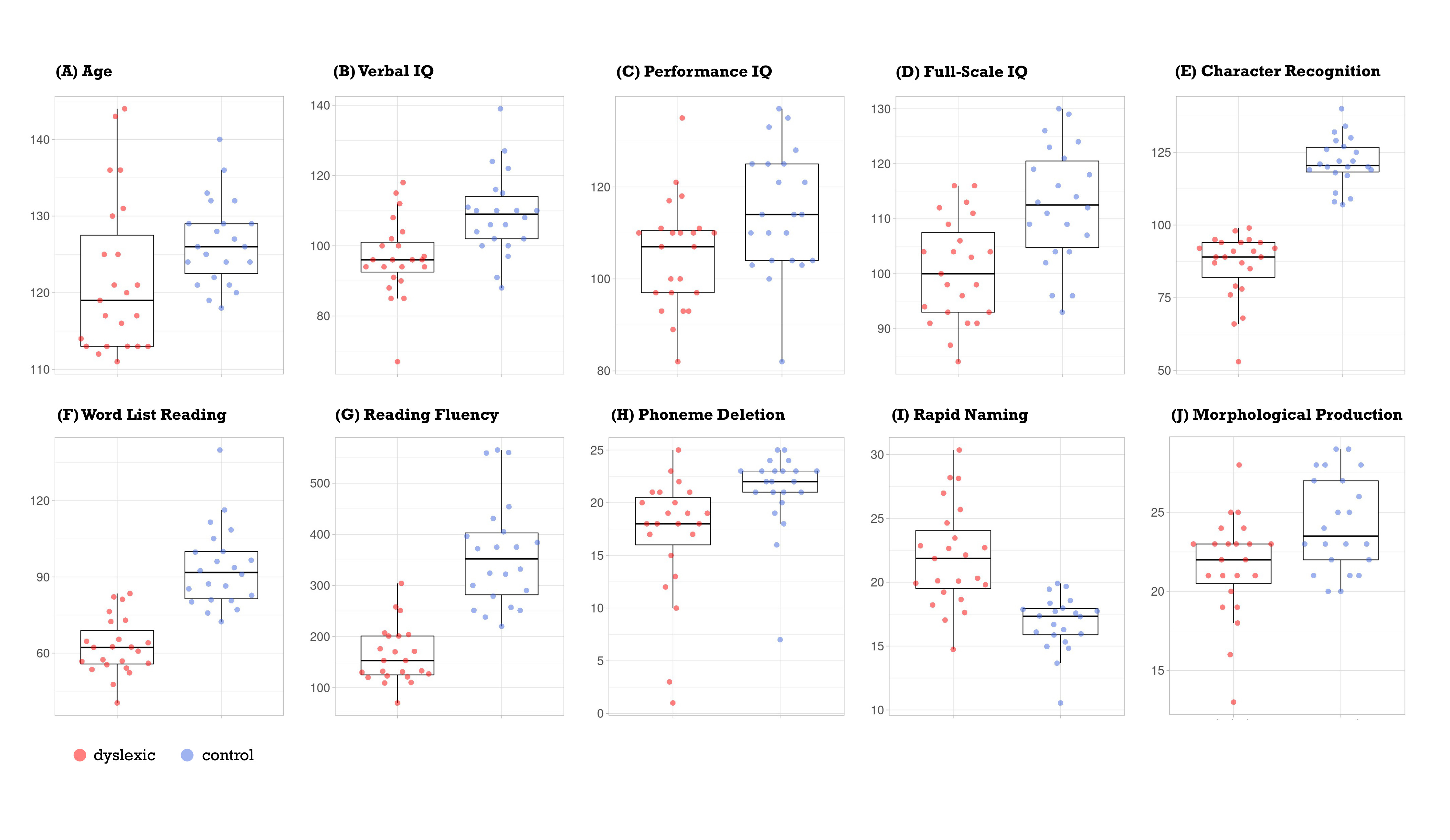

Supplement: Supplementary Figure 1 — The summary of demographic and behavioral measures is shown as boxplots, with the box indicating the IQR. The whiskers show the range of values within 1.5 × IQR and a horizontal line indicating the median. Individual data are shown as dots. The color coding is indicated in the legend below to the plot. Data visualization was performed with PlotsOfData (https://huygens.science.uva.nl/PlotsOfData/). IQR, interquartile range. [file Image_1.JPEG]
